# Determination of bacteria migration speed through urinary catheter systems in case of urostomy

**DOI:** 10.1186/2047-2994-4-S1-P219

**Published:** 2015-06-16

**Authors:** FHH Brill, H Braunwarth, D Hegeholz, W Droste

**Affiliations:** 1 Dr. Brill + Partner Gmbh Instiute for Hygiene and Microbiology, Hamburg, Germany; 2 Coloplast GmbH, Hamburg, Germany; 3 Private Consultant, Selm, Germany

## Introduction

Following a urostomy, the main aim from a hygiene perspective is to prevent bacteria from accumulating in the artificial drainage system (splint), e.g. as a result of contaminated urine. A return stop in the urostomy pouch keeps this risk to a minimum. In practice, however, splints are often pushed through the return stop to keep them more securely in place, which means that they may come into direct contact with the potentially contaminated urine.

## Objectives

The Objective was to study the migration speed of clinically-relevant bacteria in catheter systems used after urostomy.

## Methods

We carried out an in-vitro experiment in a commercially-available uriniferous system applied in a urostomy. This involved connecting two storage vessels: the first containing splints which had previously been rinsed once with artificial urine; and the second containing a bacterial suspension of the test bacteria (*E. coli, P. aeruginosa* and *P. mirabilis*), which had previously been soaked in artificial, sterile urine. The two storage vessels were inclubated at 36 °C for 24 to 72 hours. The splints were cut into segments of 5 cm after 24 hours, 48 hours and 72 hours. The colony-forming units (CFU) on the pieces were determined. Each experiment was carried out nine times before the average values and standard deviations were subsequently determined.

## Results

After 24 hours the bacteria migrated into the splint, on average, as follows: *E. coli* 26.7 cm ± 20.6, *S. aureus* 27.2 cm ± 10.6 and *P. mirabilis* 12.8 cm ± 16.2. After 48 hours the bacteria migrated as follows: 35.0 cm ± 11.2 (*E. coli*), 51.7 cm ± 7.5 (*S. aureus*) and 41.7 cm ± 23.6 (*P. mirabilis*). The results after 72 hours were: 49.4 cm ± 14.5 (*E. coli*), 60 cm ± 16.0 (S. aureus) and 67.8 cm ± 3.6 (*P. mirabilis*).

## Conclusion

The test bacteria grew relatively quickly through the catheter. It is likely that bacteria would grow through catheters with 80 cm length within a week at the latest. In this case, these is a direct infection risk for bladder and kidneys of the patient. These results should be taken into consideration during clinical use of the catheter systems in case urostomy.

## Disclosure of interest

F. Brill Grant/Research support from: partially by Coloplast; Germany., H. Braunwarth Employee of: Coloplast, Germany., D. Hegeholz Employee of: Coloplast, Germany., W. Droste: None declared.

